# Mental health of clinic-attending Syrian refugee women in Jordan: associations between social ecological risks factors and mental health symptoms

**DOI:** 10.1186/s12905-021-01584-y

**Published:** 2022-01-08

**Authors:** Mohamad Adam Brooks, Melissa Meinhart, Luma Samawi, Trena Mukherjee, Ruba Jaber, Hani Alhomsh, Neeraj Kaushal, Raeda Al Qutob, Maysa’ Khadra, Nabila El-Bassel, Anindita Dasgupta

**Affiliations:** 1 School of Social Work, Columbia University, 1255 Amsterdam Ave, New York, NY 10027 USA; 2 Department of Epidemiology, Mailman School of Public Health, Columbia University, New York, USA; 3 University of Jordan School of Medicine, Amman, Jordan

**Keywords:** Refugee, Syria, Mental health, Anxiety, Depression, PTSD, Social ecological, Jordan

## Abstract

**Background:**

The mental health of refugee women is often affected by multiple risk factors in their social ecology. Assessing these risk factors is foundational in determining potential areas for intervention. We used the social ecological model to examine risk factors associated with self-reported mental health symptoms among clinic-attending Syrian refugee women in Jordan. We hypothesize that individual (older age, unmarried, have more children under 18, difficulty reading/writing with ease), interpersonal (intimate partner violence [IPV]), community and societal level risk factors (greater number of postmigration stressors), will be associated with depression, anxiety, and post-traumatic stress disorder (PTSD) symptoms.

**Methods:**

We surveyed 507 women using a cross-sectional clinic-based systematic sampling approach between April and November 2018. We used multivariable regressions to examine associations between different risk factors in the social ecology on depression, anxiety, and PTSD. Additional multivariable regressions explored associations between specific postmigration stressors and mental health conditions.

**Results:**

We found rates of depression among our sample to be 62.92%; anxiety 57.46%; and PTSD 66.21%. Our hypothesis was partially supported. At the individual level, age was directly associated with anxiety (aOR 1.04, 95% CI [1.02, 1.06]) and PTSD (aOR 1.03, 95% CI [1.01, 1.06]), while marriage decreased odds for depression (aOR 0.41, 95% CI [0.19, 0.92]) and PTSD (aOR 0.36, 95% CI [0.15, 0.87]). IPV was associated with depression (aOR 2.78, 95% CI [1.72, 4.47]); anxiety (aOR 3.30, 95% CI [2.06, 5.27]); and PTSD (aOR 5.49, 95% CI [3.09, 9.76]). Each additional community and societal risk factor (postmigration stressor) increased the odds for depression (aOR 1.32, 95% CI [1.22, 1.42]), anxiety (aOR 1.28, 95% CI [1.19, 1.39]), and PTSD (aOR 1.46, 95% CI [1.33, 1.60]).

**Conclusion:**

Understanding social ecological risk factors associated with mental health conditions of Syrian refugee women is vital to addressing their mental health needs. IPV and postmigration stressors are consistently impactful with all mental health conditions. IPV resulted in the largest odds increase for all mental health conditions. Multilevel interventions are needed to address mental health risk factors at multiple levels of the social ecology.

## Background

Over a decade after the 2011 Syrian civil war, more than 6.7 million Syrian refugees have been forcibly displaced from their country [1]. Jordan hosts over 1.3 million registered and unregistered Syrian refugees, making it one of the largest per capita refugee hosting countries in the world [2]. Since the onset of this crisis, Jordan has stepped-up to support Syrian refugees, but has faced limited resources to meet complex health needs [2].

Syrian refugees face an increased risk for mental health conditions due to violence associated with war, issues related with adaptation to host communities, and barriers to access mental health services [3, 4]. Refugee women, moreover, face additional risk for adverse health and mental health conditions [5], as they are at greater risk of sexual, physical, and psychological abuse during displacement [6, 7]. A review of primary health needs of displaced Syrians in Jordan and other neighboring countries identified mental health and women’s health care to be the greatest needs in the region [8]. Understanding gender-specific risk factors associated with the mental health of Syrian refugees in Jordan is needed, as sex and gender differences in prevalence, symptomatology, as well as protective and risk factors for mental health have been well documented [9, 10].

The social ecological model provides a framework to understand the complex interplay between factors at multiple levels (individual, interpersonal, community, and societal), and how they can influence health outcomes [11, 12]. Based on Bronfenbrenner’s ecological theory of human development [13], the social ecological model was adapted for use with refugee populations to incorporate a more holistic approach to understand psychological distress, and as a departure from the medical model of refugee mental health [14, 15]. The social ecological model emphasizes that refugee distress is associated with prior war exposure as well as ongoing stressors in their social ecology [16]. This model has been adapted and applied to a variety of studies on refugee health and mental health in different context [17–21], and provides a framework to understand factors that places refugees at risk for mental health conditions.

Individual risk factors include biological and personal factors that increase the odds of having a mental health condition. Older age and higher education have been linked to worse mental health conditions in an earlier meta-analysis of refugees and internally displaced persons [22], but a more recent review of mental health of war refugees displaced longer than 5 years found that in some studies, lower education was a risk factor [23]. These studies suggest younger age to be associated with greater resilience and higher education linked to economic opportunity. Not being married is another individual risk factor, where among Syrian refugees in Lebanon, being married may be seen as a protective factor from physical and sexual harassment from strangers [24]. Having a husband and children(s) may also be seen as a risk factor for women, as women may prioritize the needs of their family instead of their own [24].

Interpersonal risk factors include relationships with others that can increase the odds of a mental health conditions. Existing literature highlight refugee women to be vulnerable to intimate partner violence (IPV) [21, 25], making IPV a serious health and human rights problem at the interpersonal level. IPV is estimated to affect 30% of women during their lifetime [26], and is associated with increased risk of mental health conditions [27]. A systematic review of factors associated with IPV and victimization among refugee and asylum seekers include low education level, having a nonresident legal status, and relationship discord [21]. Few have examined IPV and mental health conditions among Syrian refugees in Jordan; however, in northern Syria, IPV was associated with increased depressive symptoms among women affected by war [28].

Community and societal level risk factors include postmigration stressors, which have long been linked with mental health conditions among refugees [29–31]. Common postmigration stressors associated with mental health conditions include poverty, poor employment opportunities, inadequate housing, language barriers, challenges with asylum-seeking process, loneliness and isolation, as well as discrimination from the host community [29]. A comparison study of Syrian refugees in Turkey and New York highlight how different postmigration living difficulties impact mental health, and emphasized the importance of context-specific factors [32]. In Jordan, a qualitative synthesis of humanitarian organization supporting Syrian refugees noted postmigration stressors such as lack of income, insecure housing, employment restrictions, loss of social support to exacerbate psychological distress among Syrian refugees [33].

This paper examines the mental health service needs of 507 clinic-attending Syrian refugee women living in non-camp settings in Jordan. First, we examine the percent of our sample of Syrian refugees with and without depression, anxiety, or PTSD. Second, we examine the association between individual level risk factors, interpersonal risk factors, as well as community and societal factors on mental health conditions. Lastly, we explore specific postmigration stressors associated with mental health conditions.

We hypothesize that women who are older, unmarried, have more children under 18, have difficulty reading/writing, report IPV, experience greater number of postmigration stressors, will experience greater odds of depression, anxiety, and PTSD. Understanding social ecological risk factors associated with the mental health of Syrian refugee women is vital to addressing their mental health needs.

## Methods

### Study design and sample

We used data from Women ASPIRE, a cross-sectional study conducted between April and November 2018 that examined health inequities of 507 Syrian refugee women living in non-camp settings in Jordan. Participants were recruited from health clinics in four different cities in Jordan: Amman, Ramtha, Mafraq and Zarqa. Health clinics were selected by identifying geographic location with highest concentration of Syrian refugees living in non-camp settings in Jordan.

We recruited participants using a clinic-based systematic sampling method. Every 3^rd^ or 5^th^ (depending on clinic size) participant seeking health services was screened for eligibility. Participants were eligible if they were Syrian refugees, female, 18 years or older, did not live in a refugee camp, and did not show signs of cognitive impairment based on the Folstein Mini-Mental state exam [34]. In accordance with local customs, compensation packages of daily useable goods (valued approximately 7 USD) were provided to participants. Recruitment and surveys were completed by trained research assistants in private rooms at participant health clinics. Surveys were interviewer-administered in Arabic. Written consent was obtained from all participants. Study protocols were approved by Columbia University Institutional Review Board and Ethics Committee of University of Jordan prior to the start of the study.

### Mental Health

#### Dependent variables

**The Center for Epidemiological Studies Depression Scale (CES-D):** we used a shortened 4-item Likert scale used to measure self-reported symptoms of depression in women over the past week, translated in Arabic [35, 36]. Scores were summed to produce a score between 0 and 12. Scores of 4 or higher met the probable case for depression [35].

**The Generalized Anxiety Disorder (GAD-7):** we used a 7-item Likert scale to measure self-reported symptoms of generalized anxiety over the past 2 weeks, validated in Arabic [37, 38]. Scores were summed to produce a score between 0 and 21. Scores of 10 or higher met the probable case for generalized anxiety disorder [37].

**The PTSD Checklist for DSM-5 (PCL-5):** we used a 20-item Likert scale to measure the presence and severity of self-reported PTSD symptoms over the past 30 days, validated in Arabic [39, 40]. Scores were summed to produce a score between 0 and 80. Scores of 23 or higher met the probable case for PTSD [40].

Cronbach’s alpha estimating internal consistency among the sample was acceptable (CES-D (α = 0.79); GAD-7 (α = 0.85); and PCL-5 (α = 0.94)).

#### Independent variables

Individual, interpersonal, community and societal level mental health risk factors perceived by Syrian refugee women living in non-camp settings in Jordan were included as independent variables.

### Individual level risk factors

We included several variables such as age (continuous), marriage status (unmarried/married), number of children in household under 18 (continuous), and ability to read/write (No/Yes).

### Interpersonal level risk factors

*Intimate partner violence:* IPV was measured using the shortened 8-item Revised Conflict Tactics Scale [41]. Women were asked to report (yes/no) whether they experienced any of the six items measuring various forms of physical violence, or any of the two items measuring sexual violence in the past year. Women that responded ‘yes’ to any one of the forms of physical or sexual violence were dichotomized as having experienced intimate partner violence in the past year.

### Community and societal level risk factors

*Postmigration stressors:* Postmigration stressors were measured using the Postmigration Living Difficulties (PMLD) checklist. The PMLD is a checklist used to measure the severity of postmigration problems commonly encountered by refugees and asylum seekers [42]. Similar to previous studies [43], we used a shortened 14-item checklist that asked a range of postmigration problems related to poverty, unemployment, discrimination, separation from family members, and issues related to immigration challenges using a 6-point Likert scale. We dichotomized the PMLD scale similar to previous studies [30, 31, 42]. Participants who rated a PMLD as a Big or Very Big problem were compared to those who rated it as Not Applicable, No, Slight and Moderate problem. As each individual item measured different postmigration problems, reliability was not measured.

### Covariates

We included number of years residing in Jordan (continuous), time displaced in Syria (less than 1 month/1 month–less than 1 year/1 year or longer), and clinic location (Amman/Zarqa/Mafraq/Ramtha) as covariates in the multivariable models.

### Data analyses

Descriptive statistics were used to examine sociodemographic characteristics and mental health conditions of Syrian refugee women. Chi-square test or Fisher’s exact test was used to calculate significant differences in categorical variables between two groups, while t-test was used to calculate significant mean differences for continuous variables between two groups. Participants with a missing survey response for the mental health scales (CES-D, GAD-7, and PCL-5) were included in the data analyses if missing response did not affect cutoff scores.

We used several multivariable logistic regression models to determine the independent contribution of each social ecological risk factors on each mental health outcome. Multiple logistic regression models examined the association between individual (older age, unmarried, greater number of children under 18, difficulty reading and writing with ease), interpersonal (IPV), community and societal level risk factors (greater number of postmigration stressors) on depression, anxiety, and PTSD. Adjusted covariates include time displaced in Syria, years in Jordan, and clinic location.

Additional multiple logistic regression models were used to determine the association between specific community and societal level risk factors (postmigration stressors) on depression, anxiety, and PTSD. Specific postmigration stressors include: poverty, fears of being returned to Syria, worries about not getting treatment for health problems, worry about family in Syria, unable to return home in emergency, not being able to find work, poor access to schooling for children, loneliness and boredom, isolation, family separation, poor access to psychological services, discrimination, immigration application challenges, and communication. Of the 14 specific postmigration stressors, loneliness and boredom was omitted from the multivariable analysis due to concern of a bidirectional association with mental health conditions. Adjusted covariates include individual (age, marriage status, number of children under 18, ability to read/write) and interpersonal (IPV) risk factors, as well as time displaced in Syria, years in Jordan, and clinic location. All analyses were completed using STATA (version 15.1) [44].

## Results

### Characteristics of the participant

Characteristics of women enrolled in our study are presented in Table 1. Most women surveyed were from As-Suwayda, Daraa or Qunitra (35.9%), followed by Hama or Homs (22.7%), Aleppo or Idlib (18.9%), Damascus or Rif Dimashq (11.8%), and Al-Raqqah, Deir ez-Zor, or Hasaka (10.7%). On average, participants lived in Jordan for 5 years (SD = 1.4). Less than half (39.9%) of women were displaced in Syria for less than 1 month, with both 1 month–less than a year (29.2%) and 1 year or longer (30.9%) categories each appear in one-third of respondents. A total of 507 Syrian refugee women were surveyed from health clinics located in Amman (30.2%), Ramtha (25.3%), Mafraq (24.9%), and Zarqa (19.7%).

**Table 1:** Characteristics of social ecological risk-factors among Syrian refugee women in Jordan

		Depression (n = 507)			Anxiety (n = 503)			Post-traumatic stress disorder (n = 506)		
	Total (n = 507)	Participants not meeting depression criteria (n = 188)	Participants meeting depression criteria (n = 319)	Chi-square or T-test	Participants not meeting anxiety criteria (n = 214)	Participants meeting anxiety criteria (n = 289)	Chi-square or T-test	Participants not meeting PTSD criteria (n = 171)	Participants meeting PTSD criteria (n = 335)	Chi-square or T-test
	n(%) or ${\overline{\text{x}}}$ (SD)	n(%) or ${\overline{\text{x}}}$ (SD)	n(%) or ${\overline{\text{x}}}$ (SD)		n(%) or ${\overline{\text{x}}}$ (SD)	n(%) or ${\overline{\text{x}}}$ (SD)		n(%) or ${\overline{\text{x}}}$ (SD)	n(%) or ${\overline{\text{x}}}$ (SD)	
**Individual risk factors**										
Age (SD)	34.15 (10.99)	32.21 (10.50)	35.30 (11.13)	**p = 0.002**^a^	31.39 (9.61)	36.23 (11.46)	**p < 0.001**^a^	31.54 (10.21)	35.43 (11.11)	**p < 0.001**^a^
Marriage status (%)										
Unmarried	50 (9.86)	12 (6.38)	38 (11.91)	**p = 0.044**	15 (7.01)	34 (11.76)	p = 0.075	11 (6.43)	38 (11.34)	p = 0.077
Married	457 (90.14)	176 (93.62)	281 (88.09)		199 (92.99)	255 (88.24)		160 (93.57)	297 (88.66)	
Children under 18 in Household (SD)	3.39 (2.08)	3.40 (1.94)	3.39 (2.16)	p = 0.971^a^	3.25 (1.89)	3.52 (2.21)	p = 0.155^a^	3.14 (1.89)	3.53 (2.16)	**p = 0.0434**^a^
Ability to read and write with ease (%)										
No	439 (86.59)	166 (88.30)	273 (85.58)	p = 0.386	184 (85.98)	251 (86.85)	p = 0.778	145 (84.80)	293 (87.46)	p = 0.405
Yes	68 (13.41)	22 (11.70)	46 (14.42)		30 (14.02)	38 (13.15)		26 (15.20)	42 (12.54)	
**Interpersonal risk factors**										
Past year physical and/or sexual IPV (%)										
Yes	173 (34.12)	37 (19.68)	136 (42.63)	**p < 0.001**	43 (20.09)	129 (44.64)	**p < 0.001**	22 (12.87)	151 (45.07)	**p < 0.001**
No	334 (65.88)	151 (80.32)	183 (57.37)		171 (79.91)	160 (55.36)		149 (87.13)	184 (54.93)	
**Community and societal risk factors**										
Number of postmigration stressors (SD) n = 494	7.43 (2.98)	5.94 (2.91)	8.32 (2.65)	**p < 0.001**^a^	6.16 (3.15)	8.40 (2.44)	**p < 0.001**^a^	5.49 (2.99)	8.46 (2.41)	**p < 0.001**^a^
**Covariates**										
Years in Jordan (SD)	5.18 (1.38)	5.20 (1.38)	5.17 (1.38)	p = 0.796^a^	5.01 (1.29)	5.32 (1.42)	**p = 0.0129**^a^	5.06 (1.29)	5.24 (1.42)	p = 0.918^a^
Time displaced in Syria (SD) n = 504										
Less than 1 month	201 (39.88)	90 (48.39)	111 (34.91)		95 (44.60)	105 (36.59)		80 (47.06)	121 (36.34)	
1 month–less than 1 year	147 (29.17)	51 (27.42)	96 (30.19)	**p = 0.007**	66 (30.99)	80 (27.87)	**p = 0.026**	47 (27.65)	100 (30.03)	**p = 0.050**
1 year or longer	156 (30.95)	45 (24.19)	111 (34.91)		52 (24.41)	102 (35.54)		43 (25.29)	112 (33.63)	
Clinic location (%)										
Amman	153 (30.18)	72 (38.30)	81 (25.39)		87 (40.65)	64 (22.15)		71 (41.52)	82 (24.48)	
Zarqa	100 (19.72)	47 (25.00)	53 (16.61)	**p < 0.001**	50 (23.36)	50 (17.30)	**p < 0.001**	42 (24.56)	58 (17.31)	**p < 0.001**
Mafraq	126 (24.85)	30 (15.96)	96 (30.09)		36 (16.82)	89 (30.08)		31 (18.13)	94 (28.06)	
Ramtha	128 (25.25)	39 (20.74)	89 (27.90)		41 (19.16)	86 (29.76)		27 (15.79)	101 (30.15)	
Governorate of origin in Syria (%)										
Aleppo or Idlib	96 (18.93)	35 (18.62)	61 (19.12)		40 (18.69)	55 (19.03)		31 (18.13)	64 (19.10)	
Al-Raqqah, Deir ez-Zor, or Hasaka	54 (10.65)	32 (17.02)	22 (6.90)		35 (16.36)	18 (6.23)		30 (17.54)	24 (7.16)	
Damascus or Rif Dimashq	60 (11.83)	27 (14.36)	33 (10.34)	**p = 0.002**	27 (12.62)	33 (11.42)	**p = 0.005**	21 (12.28)	39 (11.64)	**p = 0.001**
As-Suwayda, Daraa or Qunitra	182 (35.90)	59 (31.38)	123 (38.56)		66 (30.84)	115 (39.79)		46 (26.90)	136 (40.60)	
Hama or Homs	115 (22.68)	35 (18.62)	80 (25.08)		46 (21.50)	68 (23.53)		43 (25.15)	72 (21.49)	

Chi-square test used unless otherwise noted; significant p-values (p < .05) are highlighted in bold

*SD* standard deviation

^a^T-test used

### Mental health

Most women surveyed met the criteria for a mental health condition (Table 2). We found rates of depression to be 62.92%, anxiety 57.46%, and PTSD 66.21%. CES-D scores ranged from 0 to 12 (data not shown), with almost two-thirds (62.92%) meeting the cutoff for depression (CES-D score of 4 or higher). GAD-7 scores ranged from 0 to 21 (data not shown), and over half (57.46%) met the cutoff for anxiety (GAD-7 score of 10 or higher). Lastly, PCL-5 scores ranged from 0 to 78 (data not shown), where approximately two-thirds (66.21%) met the cutoff for PTSD (PCL-5 score of 23 or higher).

**Table 2:** Cutoff scores for mental health conditions (N = 507)

	n	%
*Center for Epidemiological Studies (CES-D) 4-item Scale*	507	
Cutoff < 4	188	37.08
Cutoff ≥ 4	319	62.92
*Generalized Anxiety Disorder (GAD-7)*	503	
Cutoff < 10	214	42.54
Cutoff ≥ 10	289	57.46
*PTSD Checklist for DSM-5 (PCL-5)*	506	
Cutoff < 23	171	33.79
Cutoff ≥ 23	335	66.21

n = sample size; % = percentage

Bivariate findings of covariates and mental health conditions in Table 1 demonstrate that women with depression have significant differences in their time displaced in Syria (p = 0.007), clinic location (p < 0.001), and Syrian region of origin (p = 0.002) than compared to those without depression. Furthermore, women with anxiety had significant differences in the years in Jordan (p = 0.0129), time displaced in Syria (p = 0.026), clinic location (p < 0.001), and Syrian region of origin (p = 0.005) than compared to those without anxiety. Lastly, among women with PTSD, significant differences were found in bivariate analyses of time displaced in Syria (p = 0.050), clinic location (p < 0.001) and Syrian region of origin (p = 0.001) than compared to those without PTSD.

### Individual level risk factors

Ages ranged from 18 to 74 years old (data not shown), with a mean age of 34 years (SD = 11). Most women were married (90.1%). The number of children under 18 in the household ranged from 0 to 11 (data not shown), with an average of 3 children (SD = 2.1). Most women reported being unable to read and write with ease (86.6%), and 13.4% reported being able to do both. Women with depression, anxiety, or PTSD were, on average, significantly older than those without a mental health condition; depression (p = 0.002), anxiety (p < 0.001), or PTSD (p < 0.001). A significantly higher proportion of women with depression were unmarried (p = 0.044). We also found a higher proportion of women with anxiety and PTSD were unmarried; however, this association only trended towards statistical significance. Women with PTSD had, on average, more children under 18 living in the household than compared to those without PTSD (p = 0.0434). We did not find any significant association between depression or anxiety and the number of children under 18 living in the household.

### Interpersonal level risk factors

Approximately a third of women surveyed (34.1%) reported IPV during the past year. A significantly higher proportion of women with depression (p < 0.001), anxiety (p < 0.001), or PTSD (p < 0.001) reported IPV during the past year than compared to those who did not meet mental health conditions.

### Community and Societal Level Risk Factors

Table 3 provides findings between postmigration stressors and mental health conditions. Women, on average, experienced 7.43 (SD = 2.98) postmigration stressors (Table 1). Women with depression, anxiety, or PTSD experienced, on average, a greater number of postmigration stressors (p < 0.001) than compared to women without a mental health condition. Out of the 14 postmigration stressors asked (Table 3), the top three identified as being a big/very big stressor were: poverty (79.1%), fears of being forced to return to Syria (78.9%) and worries about not getting treatment for health problems (74.5%). The three least reported postmigration stressors were: discrimination from local non-Syrian community (16.2%), immigration application challenges (15.8%), and communication with local non-Syrian community (6.3%).

**Table 3:** Bivariate associations between specific postmigration stressors (PMLDs) on depression, anxiety and PTSD

		Depression (n = 507)			Anxiety (n = 503)			Post-traumatic stress disorder (n = 506)		
	Total (n = 507)	Participants not meeting depression criteria (n = 188)	Participants meeting depression criteria (n = 319)	Chi-square or Fisher’s exact test	Participants not meeting anxiety criteria (n = 214)	Participants meeting anxiety criteria (n = 289)	Chi-square or Fisher’s exact test	Participants not meeting PTSD criteria (n = 171)	Participants meeting PTSD criteria (n = 335)	Chi-square or Fisher’s exact test
	n(%) or ${\overline{\text{x}}}$ (SD)	n(%) or ${\overline{\text{x}}}$ (SD)	n(%) or ${\overline{\text{x}}}$ (SD)		n(%) or ${\overline{\text{x}}}$ (SD)	n(%) or ${\overline{\text{x}}}$ (SD)		n(%) or ${\overline{\text{x}}}$ (SD)	n(%) or ${\overline{\text{x}}}$ (SD)	
Poverty (%)										
Big/very big problem	401 (79.09)	125 (66.49)	276 (86.52)	**p < 0.001**	146 (68.22)	252 (87.20)	**p < 0.001**	108 (63.16)	293 (87.46)	**p < 0.001**
No/moderate problem	106 (20.91)	63 (33.51)	43 (13.48)		68 (31.78)	37 (12.80)		63 (36.84)	42 (12.54)	
Fears of forced return to Syria (%) n = 506										
Big/very big problem	399 (78.85)	145 (77.13)	254 (79.87)	p < 0.465	164 (76.64)	231 (80.21)	p < 0.334	123 (71.93)	275 (82.34)	**p = 0.007**
No/moderate problem	107 (21.15)	43 (22.87)	64 (20.13)		50 (23.36)	57 (19.79)		48 (28.07)	59 (17.66)	
Worries about not getting treatment for health problem (%) n = 506										
Big/very big problem	377 (74.51)	120 (63.83)	257 (80.82)	**p < 0.001**	139 (65.26)	236 (81.66)	**p < 0.001**	99 (57.89)	278 (83.23)	**p < 0.001**
No/moderate problem	129 (25.49)	68 (36.17)	61 (19.18)		74 (34.74)	53 (18.34)		72 (42.11)	56 (16.77)	
Worry of family in Syria (%)										
Big/very big problem	372 (73.37)	119 (63.30)	253 (79.31)	**p < 0.001**	135 (63.08)	235 (81.31)	**p < 0.001**	99 (57.89)	273 (81.49)	**p < 0.001**
No/moderate problem	135 (26.63)	69 (36.70)	66 (20.69)		79 (36.92)	54 (18.69)		72 (42.11)	62 (18.51)	
Unable to return home in emergency (%) n = 506										
Big/very big problem	365 (72.13)	114 (60.64)	251 (78.93)	**p < 0.001**	132 (61.68)	230 (79.86)	**p < 0.001**	93 (54.39)	272 (81.44)	**p < 0.001**
No/moderate problem	141 (27.87)	74 (39.36)	67 (21.07)		82 (38.32)	58 (20.14)		78 (45.61)	62 (18.56)	
Not being able to find work (%) n = 504										
Big/very big problem	361 (71.63)	119 (63.30)	242 (76.58)	**p = 0.001**	136 (63.55)	224 (78.32)	**p < 0.001**	103 (60.59)	258 (77.48)	**p < 0.001**
No/moderate problem	143 (28.37)	69 (36.70)	74 (23.42)		78 (36.45)	62 (21.68)		67 (39.41)	75 (22.52)	
Poor access to schooling for children (%)										
Big/very big problem	318 (62.72)	101 (53.72)	217 (68.03)	**p = 0.001**	118 (55.14)	199 (86.78)	**p = 0.002**	81 (47.37)	237 (70.75)	**p < 0.001**
No/moderate Problem	189 (37.28)	87 (46.28)	102 (31.97)		96 (44.86)	90 (31.14)		90 (52.63)	98 (29.25)	
Loneliness and boredom (%) n = 506										
Big/very big problem	289 (57.11)	61 (32.45)	228 (71.70)	**p < 0.001**	83 (38.79)	203 (70.49)	**p < 0.001**	55 (32.16)	234 (70.06)	**p < 0.001**
No/moderate problem	217 (42.89)	127 (67.55)	90 (28.30)		131 (61.21)	85 (29.51)		116 (67.84)	100 (29.94)	
Isolation (%) n = 503										
Big/very big problem	251 (49.90)	54 (29.03)	197 (62.15)	**p < 0.001**	73 (34.27)	176 (61.54)	**p < 0.001**	52 (30.41)	199 (60.12)	**p < 0.001**
No/moderate problem	252 (50.10)	132 (70.97)	120 (37.85)		140 (65.73)	110 (38.46)		119 (69.59)	132 (39.88)	
Family separation (%)										
Big/very big problem	248 (48.92)	72 (38.30)	176 (55.17)	**p < 0.001**	84 (39.25)	162 (56.06)	**p < 0.001**	61 (35.67)	187 (55.82)	**p < 0.001**
No/moderate problem	259 (51.08)	116 (61.70)	143 (44.83)		130 (60.75)	127 (43.94)		110 (64.33)	148 (44.18)	
Poor access to psychological services (%)										
Big/very big problem	194 (38.26)	41 (21.81)	153 (47.96)	**p < 0.001**	53 (24.77)	141 (48.79)	**p < 0.001**	34 (19.88)	160 (47.76)	**p < 0.001**
No/moderate problem	313 (61.74)	147 (78.19)	166 (52.04)		161 (75.23)	148 (51.21)		137 (80.12)	175 (52.24)	
Discrimination (%) n = 506										
Big/very big problem	82 (16.17)	14 (7.45)	68 (21.32)	**p < 0.001**	20 (9.35)	59 (20.42)	**p = 0.001**	9 (5.29)	73 (21.79)	**p < 0.001**
No/moderate problem	425 (83.83)	174 (92.55)	251 (78.68)		194 (90.65)	229 (79.58)		162 (94.74)	262 (78.21)	
Immigration application challenges (%) n = 505										
Big/very big problem	80 (15.84)	23 (12.30)	57 (17.92)	p = 0.095	31 (14.55)	49 (17.01)	p = 0.457	18 (10.59)	62 (18.56)	**p = 0.021**
No/moderate problem	425 (84.16)	164 (87.70)	261 (82.08)		182 (85.45)	239 (82.99)		152 (89.41)	272 (81.44)	
Communication (%) n = 506										
Big/very big problem	32 (6.32)	7 (3.74)	25 (7.84)	p = 0.068	7 (3.29)	25 (8.65)	**p = 0.015**	1 (0.59)	31 (9.25)	**p < 0.001**^a^
No/moderate problem	474 (93.68)	180 (96.26)	294 (92.16)		206 (96.71)	264 (91.35)		169 (99.41)	304 (90.75)	

PMLDs are listed according to greatest frequency of being a Big/Very Big Problem; Chi-square test used unless otherwise noted; significant p-values (p < .05) are highlighted in bold

*SD* standard deviation

^a^Fisher’s exact test used

Table 3 provides bivariate findings between the severity of postmigration stressors (big/very big problem or no/moderate problem) among those with mental health conditions and those without. In the case of 11 of the 14 postmigration stressors, among women with depression, anxiety, or PTSD, the proportion of those who perceived postmigration stressors as a big/very big problem was significantly larger than compared to women without depression, anxiety, or PTSD. This included 11 postmigration stressors for depression (range: p < 0.001 to p = 0.001), 12 postmigration stressors for anxiety (range: p < 0.001 to p = 0.015), and all 14 postmigration stressors for PTSD (range: p < 0.001 to p = 0.021).

### Associations between social ecological risk factors and mental health

We found certain risk factors in the social ecology to increase the odds of mental health conditions than compared to others. Table 4 provides multivariable findings between individual, interpersonal, community and societal level risk factors on mental health conditions. Among the individual level risk factor, we found that each additional year of age was significantly associated with a 4% (between 2 and 6% or 7%) increased odds of anxiety in the unadjusted (OR 1.04, 95% CI [1.02, 1.07]) and adjusted (aOR 1.04, 95% CI [1.02, 1.06]) model, as well as an 3% or 4% (between 1 and 6%) increased odds of PTSD in the unadjusted (OR 1.04, 95% CI [1.01, 1.06]) and adjusted (aOR 1.03, 95% CI [1.01, 1.06]) model. We also found that women who were married had a 65% (between 22 and 84%) decreased odds of depression in the unadjusted (OR 0.35, 95% CI [0.16, 0.78]) and a 59% (between 8 and 81%) decreased odds in the adjusted (aOR 0.41, 95% CI [0.19, 0.92]) model. Women who were married also had a 64% or 66% (between 13% or 19–85%) decreased odds of PTSD in the unadjusted (OR 0.34, 95% CI [0.15, 0.81]) and adjusted (aOR 0.36, 95% CI [0.15, 0.87]) model. The similarities between the unadjusted and adjusted significant values for age suggests that the adjusted covariates (years in Jordan, time displaced in Syria, and clinic location) had little/no impact on the odds of anxiety and PTSD, while for women who were married, the adjusted covariates only slightly increased the odds for depression and PTSD. We did not find associations between having more children under 18, difficulty to read and write with ease and mental health conditions.

**Table 4:** Multivariable associations between social ecological risk-factors on depression, anxiety, and PTSD

	Depression (n = 507)		Anxiety (n = 503)		Post-traumatic stress disorder (n = 506)	
	uOR (95% CI)	aOR^a^ (95% CI)	uOR (95% CI)	aOR^a^ (95% CI)	uOR (95% CI)	aOR^a^ (95% CI)
**Individual risk factors**						
Age	1.02 (1.00, 1.04)	1.01 (0.99, 1.03)	1.04 (1.02, 1.07)***	1.04 (1.02, 1.06)***	1.04 (1.01, 1.06)**	1.03 (1.01, 1.06)**
Marriage status						
Unmarried (ref.)	–	–	–	–	–	–
Married	0.35 (0.16, 0.78)**	0.41 (0.19, 0.92)*	0.49 (0.23, 1.02)	0.56 (0.26, 1.19)	0.34 (0.15, 0.81)*	0.36 (0.15, 0.87)*
Number of children < 18	0.96 (0.87, 1.06)	0.95 (0.86, 1.06)	1.07 (0.96, 1.18)	1.06 (0.96, 1.18)	1.11 (0.99, 1.25)	1.12 (0.99, 1.26)
Ability to read/write with ease						
No (ref.)	–	–	–	–	–	–
Yes	1.42 (0.75, 2.71)	1.42 (0.74, 2.73)	0.94 (0.51, 1.74)	0.97 (0.52, 1.82)	0.83 (0.42, 1.65)	0.83 (0.41, 1.67)
**Interpersonal risk factors**						
Past year physical and/or sexual IPV						
No (ref.)	–	–	–	–	–	–
Yes	2.87 (1.80, 4.58)***	2.78 (1.72, 4.47)***	3.30 (2.09, 5.20)***	3.30 (2.06, 5.27)***	5.36 (3.07, 9.38)*	5.49 (3.09, 9.76)***
**Community and societal risk factors**						
Number of postmigration stressors	1.33 (1.23, 1.43)***	1.32 (1.22, 1.42)***	1.28 (1.19, 1.39)***	1.28 (1.19, 1.39)***	1.46 (1.33, 1.60)***	1.46 (1.33, 1.60)***

The adjusted covariates were years in Jordan, time displaced in Syria, and clinic location

*CI* confidence interval, *uOR* unadjusted odds ratio, *aOR* adjusted odds ratio

Significant p-values are highlighted in bold; *p < .05; **p < .01; ***p < .001

In the interpersonal level risk factor, we found that IPV during the past year to be significantly associated with all mental health conditions. IPV was associated with a 187% (between 80 and 358%) increased odds of depression in the unadjusted model (OR 2.87, 95% CI [1.80, 4.58]) and a 178% (between 72 and 347%) increased odds in depression in the adjusted (aOR 2.78, 95% CI [1.72, 4.47]) model. IPV was associated with a 230% (between 109 and 420%) increased odds of anxiety in the unadjusted (OR 3.30, 95% CI [2.09, 5.20]) and a 230% (between 106 and 437%) increased odds in the adjusted (aOR 3.30, 95% CI [2.06, 5.27]) model. Lastly, IPV was associated with a 436% (between 207 and 838%) increased odds of PTSD in unadjusted model (OR 5.36, 95% CI [3.07, 9.38]) and a 449% (between 209 and 876%) increase odds in the adjusted (aOR 5.49, 95% CI [3.09, 9.76]) model. We found that with IPV, the adjusted covariates slightly decreased the odds for depression, but increased the odds for PTSD, and had little/no impact on the odds for anxiety.

In the community and societal risk factors, we also found that each additional postmigration stressor was significantly associated with increased odds of mental health conditions, including a 33% (between 23 and 43%) increased odds of depression in the unadjusted model (OR 1.33, 95% CI [1.23, 1.43]) and a 32% (between 22 and 42%) in the adjusted (aOR 1.32, 95% CI [1.22, 1.42]) model. Each additional postmigration stressor was associated with a 28% (between 19 and 39%) increased odds of anxiety in unadjusted (OR 1.28, 95% CI [1.19, 1.39]) and adjusted (aOR 1.28, 95% CI [1.19, 1.39]) model, and a 46% (between 33%-60%) increased odds of PTSD in unadjusted (OR 1.46, 95% CI [1.33, 1.60]) and adjusted (aOR 1.46, 95% CI [1.33, 1.60]) model. The similarities between the unadjusted and adjusted significant values for postmigration stressor suggesting that the adjusted covariates had little/no impact on the odds of anxiety and PTSD.

### Associations between specific community and societal level risk factors and mental health

Table 5 provides multivariable findings between specific community and societal level risk factors (postmigration stressors) on mental health conditions. Among our sample, experience of isolation as a big/very big problem was significantly associated with a 159% (between 67 and 301%) increased odds of depression in the unadjusted model (OR 2.59, 95% CI [1.67, 4.01]) as well as a 158% (between 63 and 309%) increased odds in the adjusted model (aOR 2.58, 95% CI [1.63, 4.09]); a 95% (between 20 and 195%) increased odds of anxiety in the unadjusted model (OR 1.95, 95% CI [1.28, 2.97]) and a 89% (between 20 and 199%) in the adjusted model (aOR 1.89, 95% CI [1.20, 2.99]); and a 64% (between 4 and 160%) increased odds of PTSD in the unadjusted model only (OR 1.64, 95% CI [1.04, 2.60]). We also found poor access to psychological services as a big/very big problem to have a 99% (between 24 and 290%) increased odds of depression in the unadjusted model (OR 1.99, 95% CI [1.24, 3.90]) and a 71% (between 5 and 180%) increased odds in the adjusted model (aOR 1.71, 95% CI [1.05, 2.80]). However, we found a 70% (between 9 and 164%) increased odds of anxiety in the unadjusted model (OR 1.70, 95% CI [1.09, 2.64]) and a 77% (between 8 and 192%) increased odds of PTSD (OR 1.77, 95% CI [1.08, 2.92]) in the unadjusted model only. As we did not find significant associations in the adjusted values between isolation as a big/very big problem and PTSD, as well as poor access to psychological services as a big/very big problem and anxiety or PTSD, this suggests that the adjusted covariates impacted the significance of these mental health conditions.

**Table 5:** Multivariable associations between specific community and societal-level risk factors on depression, anxiety, and PTSD

	Depression (n = 507)		Anxiety (n = 503)		Post-traumatic stress disorder (n = 506)	
	uOR (95% CI)	aOR^a^ (95% CI)	uOR (95% CI)	aOR^a^ (95% CI)	uOR (95% CI)	aOR^a^ (95% CI)
Poverty	1.57 (0.93, 2.66)	1.45 (0.82, 2.54)	1.69 (1.00, 2.87)	1.36 (0.76, 2.45)	1.84 (1.07, 3.16)*	1.45 (0.79, 2.67)
Fears of forced return to Syria	0.67 (0.39, 1.14)	0.58 (0.32, 1.05)	0.70 (0.42, 1.18)	0.73 (0.41, 1.29)	0.84 (0.49, 1.46)	0.86 (0.46, 1.63)
Worries about not getting treatment for health problem	1.38 (0.83, 2.28)	1.28 (0.75, 2.21)	1.39 (0.84, 2.29)	1.26 (0.73, 2.18)	1.89 (1.12, 3.16)*	2.05 (1.14, 3.68)*
Worry of family in Syria	1.15 (0.69, 1.91)	1.16 (0.67, 1.99)	1.43 (0.87, 2.35)	1.43 (0.83, 2.47)	1.52 (0.90, 2.55)	1.58 (0.88, 2.85)
Unable to Return home in Emergency	1.36 (0.82, 2.25)	1.44 (0.84, 2.48)	1.36 (0.83, 2.25)	1.29 (0.75, 2.23)	1.86 (1.11, 3.12)*	1.98 (1.11, 3.54)*
Not being able to find work	1.08 (0.67, 1.73)	1.06 (0.64, 1.76)	1.24 (0.78, 1.96)	1.27 (0.76, 2.11)	1.10 (0.68, 1.80)	1.13 (0.65, 1.95)
Poor access to schooling for children	1.03 (0.66, 1.60)	1.06 (0.65, 1.73)	1.13 (0.73, 1.73)	1.20 (0.73, 1.96)	1.45 (0.92, 2.27)	1.41 (0.84, 2.39)
Isolation	2.59 (1.67, 4.01)***	2.58 (1.63, 4.09)***	1.95 (1.28, 2.97)**	1.89 (1.20, 2.99)**	1.64 (1.04, 2.60)*	1.55 (0.93, 2.58)
Family separation	1.08 (0.69, 1.69)	1.05 (0.65, 1.70)	1.07 (0.69, 1.65)	1.13 (0.70, 1.80)	1.09 (0.68, 1.74)	1.14 (0.68, 1.93)
Poor access to psychological services	1.99 (1.24, 3.90)**	1.71 (1.05, 2.80)*	1.70 (1.09, 2.64)*	1.49 (0.92, 2.41)	1.77 (1.08, 2.92)*	1.49 (0.87, 2.57)
Discrimination	1.91 (0.97, 3.77)	2.02 (0.98, 4.14)	1.40 (0.76, 2.60)	1.55 (0.79, 3.06)	2.68 (1.24, 5.81)*	3.58 (1.56, 8.20)**
Immigration application challenges	1.27 (0.71, 2.25)	1.29 (0.69, 2.39)	0.96 (0.54, 1.65)	0.86 (0.48, 1.56)	1.48 (0.79, 2.75)	1.37 (0.68, 2.75)
Communication	0.95 (0.36, 2.51)	1.00 (0.36, 2.82)	1.46 (0.57, 3.76)	1.68 (0.61, 4.63)	N/A	N/A

The adjusted covariates were age, marriage status, ability to read and write with ease, number of children under 18, IPV past year, years in Jordan, time displaced in Syria, and clinic location

*CI* confidence interval, *uOR* unadjusted odds ratio, *aOR* adjusted odds ratio

Significant p-values are highlighted in bold; *p < .05; **p < .01; ***p < .001

We found the experience of not receiving treatment for health problems as a big/very big problem was significantly associated with a 89% (between 12 and 216%) increased odds of PTSD in the unadjusted (OR 1.89, 95% CI [1.12, 3.16]) and a 105% (14–268%) increased odds in the adjusted model (aOR 2.05, 95% CI [1.14, 3.68]). We also found being unable to return home in emergency as a big/very big problem was significantly associated with a 86% (between 11 and 212%) increased odds of PTSD in the unadjusted model (OR 1.86, 95% CI [1.11, 3.12]) and a 98% (between 11 and 254%) increased odds in the adjusted model (aOR 1.98, 95% CI [1.11, 3.54]). Discrimination as a big/very big problem was also significantly associated with an 168% (between 24 and 481%) increased odds of PTSD in the unadjusted model (OR 2.68, 95% CI [1.24, 5.81]) and a 258% (between 56 and 720%) increased odds in the adjusted model (aOR 3.58, 95% CI [1.56, 8.20]). Poverty as a big/very big problem was significantly associated with a 84% (between 7 and 216%) increased odds of PTSD in the unadjusted model only (OR 1.84, 95% CI [1.07, 3.16]). The similarities between the unadjusted and adjusted significant values for health problems, being able to return home in emergency, and discrimination as a big/very big problem, suggests that the adjusted covariates increased the odds of the significant mental health conditions. Poverty as a big/very big problem was not significant in the adjusted model, suggesting that the adjusted covariates contributed to this loss of significance.

## Discussion

**Fig. 1:**
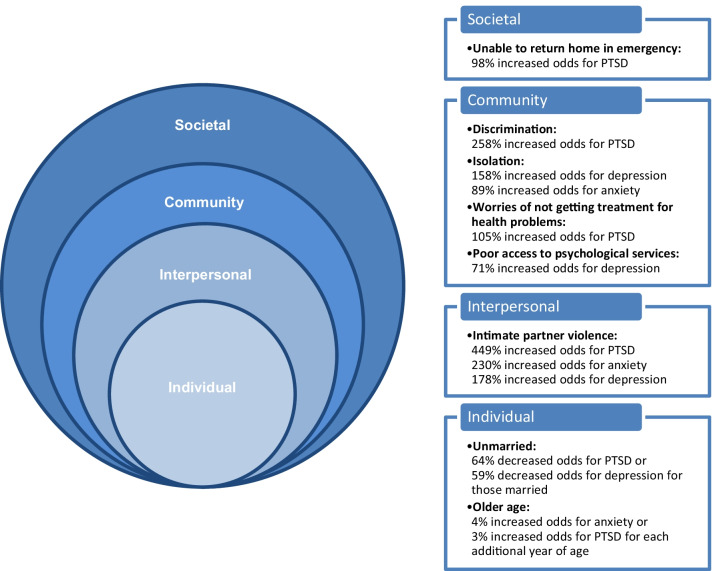
Adapted from: The Social-Ecological Model. A Framework for Prevention |Violence Prevention|Injury Center|CDC [Internet]. 2021 [cited 2021 Jul 21]. Available from: https://www.cdc.gov/violenceprevention/publichealthissue/social-ecologicalmodel.html

Our study provides an opportunity to examine the mental health of clinic-attending Syrian refugee women living in non-camp settings in Jordan. We found our hypothesis to be partially supported, in that certain (not all) risk factors from the individual, interpersonal, community and societal level risk factors increase odds of mental health conditions. We found IPV and postmigration stressors to be consistently impactful with all mental health conditions, and that IPV resulted in the largest odds increase for all mental health conditions. Figure 1 provides a visual representation of the social ecological risk factors we found for depression, anxiety, and PTSD.

We found rates of mental health conditions among our sample of Syrian refugees to be high (depression 62.92%; anxiety 57.46%; and PTSD 66.21%) when comparing them to rates from a recent systematic review of Syrian refugees displaced in 10 different countries globally: depression (20–44.1%), anxiety (19.3–31.8%), and PTSD (23.4–83.4%) [45]. One possible explanation for these high rates is because our sample focused exclusively on women, where refugee women experience additional risk to mental health conditions as a result of greater risk of sexual, physical, and psychological abuse during displacement [5–7]. Female gender was also suggested as a potential risk factor for PTSD in a study of Syrian refugees residing in a tent city in Turkey. The study found the rate of PTSD among their sample (of men and women) to be 33.5%, but that the probability of being diagnosed with PTSD was 71% if they were female gender, diagnosed with a psychiatric disorder in the past, having a family history of psychiatric disorder, and experiencing 2 or more traumas [46]. Another possible explanation for this high rate is the contextual factors faced by Syrian refugees in different countries. Syrian refugees resettled in resource-strained countries face a different set of postmigration stressors than compared to those resettled in resource-rich countries.

In the individual level, we found married women had 64% decreased odds of PTSD and 59% decreased odds of depression, while each year increase of age to be associated with 4% increased odds of anxiety and 3% increased odds of PTSD. We did not find associations between having more children under 18 and difficulty in reading/writing on mental health conditions. One explanation is that younger adult refugees may be able to better adapt and engage easier with their new host community compared to older women. Children might also provide a hope for the future. Furthermore, marriage may be a protective factor from physical and sexual harassment from strangers and provide social support [24].

We found IPV (interpersonal level) to result in the largest odds increase for depression, anxiety, or PTSD when compared to any other mental health risk factors in the social ecology. IPV resulted in a 178%-449% increased odds of experiencing a mental health condition (Table 4). Over one-third of women in our sample experienced IPV in the past year, and previous literature reported violence against Syrian refugee women and girls as an ongoing issue [47, 48]. Similar findings are also reflected in a systematic review of mental health conditions and IPV, where strong associations between IPV and depression, anxiety and PTSD were found [49]. Gender-based violence among female refugees is most-likely underreported [47], which emphasize the necessity for service providers, healthcare agencies, and policy makers to tackle IPV as public health and a human rights crisis.

At the community and societal level, we found that each additional postmigration stressor increased the odds of experiencing a mental health condition by 28–46%. This echoes similar findings in existing literature, where a strong association between postmigration stressors and mental health conditions have been highlighted [32, 33, 43]. These findings also highlight how necessary it is for service providers, healthcare agencies, and policy makers to address postmigration stressors when alleviating mental health conditions.

Among the 14 postmigration stressors we examined in our sample of refugee women, we found isolation (158% increased odds) to have the greatest odds increase for depression, followed by poor access to psychological services (71% increased odds). For anxiety, isolation (89% increased odds) was the only significant postmigration stressor in the adjusted model. We also found discrimination (258% increased odds) to have the greatest odds increase for PTSD, followed by worries of not getting treatment for health problems (105% increased odds), and unable to return home in emergency (98% increased odds). Other studies have found different postmigration stressors to be associated with mental health conditions [29–31], which may suggests an association with context- or country-specific factors. For example, a study of contextual factors and psychosocial wellbeing of Syrian refugees in Turkey and the United States found that the most pressing postmigration stressors to be related to employment and poverty in the Turkey sample, while communication difficulties, isolation, and boredom were found in the United States sample [32].

One reason why isolation had greater odds for depression and anxiety is because of the buffering effect a social network and social support may have on mental health. Previous studies have highlighted the interconnectedness between social networks, social support and psychological wellbeing among refugees [16, 50]. Service providers and healthcare agencies may benefit from facilitating a social or activity group for Syrian refugee women to reduce isolation and increase social support.

A reason why poor access to psychological services may be associated with depression is that it may represent multiple barriers faced when seeking psychological assistance. A 2020 report by International Medical Corps in Jordan identifies several barriers Syrian refugees face when seeking psychological assistance, which include hopelessness, lack of financial means, poor recognition of mental health problems, cost of treatment, need for privacy, and stigma [4]. These barriers may lead to worsening mental health symptoms due to untreated mental health conditions. Healthcare agencies can benefit from identifying and mitigating these barriers to psychological services.

We also found significant association between discrimination from local non-Syrian community and PTSD, which is supported in the literature, as discrimination experienced by refugees is associated with poor mental health conditions [51, 52]. Lastly, one possible reason why we found an association between not getting treatment for health conditions and unable to return home in emergency on PTSD may be related to the participants trauma history and worries that family in Syria might experience a similar traumatic event too.

Longitudinal studies can provide greater insight between these specific postmigration stressors and mental health conditions. For example, a study of traumatized refugee and asylum seekers in Switzerland found certain postmigration stressors to be associated with anxiety and depression, but less so with PTSD, which appear to be associated with trauma [30]. Our findings, nevertheless, highlight the need to incorporate a social ecological framework when understanding the mental health service needs of refugees.

## Limitations

The above analysis presents with the following limitations. The cross-sectional design of our study limits our ability to draw causal inferences. Mental health was self-reported rather than diagnosed by a clinician. Wide confidence intervals found in several of our significant findings suggests that a larger sample size may be needed to narrow confidence intervals and to improve the accuracy of our findings. We attempted to reduce social desirability bias throughout our study by providing comprehensive trainings for research assistant and providing a safe environment for participants. Our sample of Syrian refugees only include women who accessed services in health clinics, which limits our ability to generalize results.

## Conclusion

It is necessary that healthcare providers, governmental and non-governmental organizations (NGOs), and policy makers in Jordan be aware that the mental health of Syrian refugees in Jordan is interconnected with risk factors in multiple levels of the social ecology. A multilevel approach to mental health intervention is necessary, where in addition to direct clinical treatment by a psychologist or psychiatrist to address mental health conditions, incorporating social workers or case workers who assist with multiple post-migration stressors, and having policy makers advocate for policy-level changes, is needed to address the mental health of Syrian refugee women. These key players must also work collaboratively—and not in silos—to improve the mental health status of Syrian refugees.

It is also necessary that policy makers and healthcare agencies serving Syrian refugee women to promote greater awareness of and screening for mental health conditions, and to incorporate or refer to services appropriate for mental health. Mandatory screening tools that screen for mental health conditions and IPV must be incorporated into health clinics, as clinics are often the first line of contact for many Syrian refugee women seeking help. Furthermore, referrals must be realistic for the client and be cognizant of the barriers of care that exist for many Syrian refugee women. As mental health is often associated with stigma and discrimination, awareness of services and normalizing treatment for mental health conditions is essential.

It is necessary that future research include qualitative data so specific mechanisms on how specific postmigration living difficulties influence mental health are better understood. For example, identifying major barriers that result to poor access to psychological care is essential to figuring out how to address this issue. Lastly, longitudinal research is needed to examine how postmigration living difficulties influence mental health over time.

## Data Availability

The datasets generated and/or analyzed during the current study are not publicly available due to the sensitivity of the context and the fact that this is data pertaining to refugees. Datasets remain available from the corresponding author upon reasonable request.

## Abbreviations

aOR: Adjusted odds ratio
ASPIRE: Advancing solutions in policy, implementation, research, and engagement for refugees
CES-D: Center for epidemiological studies depression scale
CI: Confidence Interval
GAD-7: Generalized anxiety disorder scale
IPV: Intimate partner violence
OR: Odds ratio
PCL-5: The PTSD checklist for DSM-5
PMLD: Postmigration living difficulty checklist
PTSD: Post-traumatic stress disorder
SD: Standard deviation
USD: United States Dollar
